# Mn-doped ZnO nanopowders prepared by sol–gel and microwave-assisted sol–gel methods and their photocatalytic properties

**DOI:** 10.3762/bjnano.15.104

**Published:** 2024-10-28

**Authors:** Cristina Maria Vlăduț, Crina Anastasescu, Silviu Preda, Oana Catalina Mocioiu, Simona Petrescu, Jeanina Pandele-Cusu, Dana Culita, Veronica Bratan, Ioan Balint, Maria Zaharescu

**Affiliations:** 1 Institute of Physical Chemistry ‘‘Ilie Murgulescu’’ of the Romanian Academy, 202 Splaiul Independentei, 060021 Bucharest, Romania

**Keywords:** microwave-assisted synthesis, oxalic acid mineralization, semiconductor photocatalysts, water depollution

## Abstract

Although the microwave-assisted sol–gel method is quite frequently used for the preparation of oxide nanostructures, the synergism of the reaction pathways is not fully explained. However, state-of-the-art theoretical and practical results of high novelty can be achieved by continuously evaluating the as-synthesized materials. The present paper presents a comparative study of Mn-doped ZnO nanopowders prepared by both sol–gel and microwave-assisted sol–gel methods. The structural, morphological, and optical properties of the as-obtained powders were established and correlated with their newly proved functionality, namely, the ability to photogenerate distinct reactive oxygen species (·OH or O_2_^−^) and to act as photoactive materials in aqueous media. The solar light-induced mineralization of oxalic acid by Mn-doped ZnO materials was clearly observed while similar amounts of generated CO_2_ were measured for both catalysts. These inexpensive semiconductor materials, which proved to be light-responsive, can be further used for developing water depollution technologies based on solar light energy.

## Introduction

Over the past decades, significant research has been focused on designing and preparing nanostructures of various shapes and sizes, which exhibit unique properties and potential applications [1]. Considerable advancements have been made in synthesizing nanostructured materials from liquid, solid, and gaseous precursors [2]. Nonetheless, many of these methods are constrained by the necessity of high temperatures and pressures, lengthy reaction times, and toxic reagents [3–4].

Although physical methods offer high reproducibility, chemical methods are often preferred for synthesizing oxide nanostructures because of their advantages, such as uniform mixing of precursors at the molecular level, low operating temperatures, and the ability to control the physicochemical properties of the final products [5–6]. Among various chemical techniques, the sol–gel method (SG) has gained increasing prominence in materials science because of its versatility and its capacity to produce homogeneous products with high purity. Additionally, it facilitates the incorporation of dopants in significant quantities, which enables precise control over the shape and size distribution of the resulting nanomaterials [7–9].

Recently, the use of microwave energy for synthesizing functional nanomaterials has garnered significant interest [10–11]. The microwave-assisted sol–gel technique (MW) has been reported to be simpler, faster, more cost-effective, and more energy-efficient than conventional heating methods [12–14]. This approach is particularly advantageous in technology applications, as it significantly reduces reaction times from days to mere hours or minutes and enables the production of nanocrystalline oxides in the form of powders and films on various substrates [3,15–16].

While traditional heating methods cause inhomogeneities by slowly distributing the heat from the surface to the core of the material or within the entire volume of the solution, microwaves allow for quick and uniform heating because they can penetrate to a depth depending on the dielectric properties of material [17]. Therefore, instantaneous decomposition of the precursors occurs, leading to the formation of a supersaturated solution. Consequently, appropriate conditions for obtaining materials with well-defined properties (i.e., monodispersed nanoparticles from rapid and brief nucleation in a supersaturated solution) can be experimentally identified [18] and correlated with specific parameters of the systems exposed to microwaves [18–20]. For instance, the use of microwaves in nanoparticle production may increase the reaction rate, also contributing to a narrow particle size distribution, improved purity, tailored shape, and morphological uniformity [21]. More crucially, the crystallization can be influenced by varying reaction temperature, reaction duration, and system composition [18,22].

According to the literature, MW has been predominantly employed for the thermal treatment of amorphous oxide nanopowders, the precipitation of nanocrystalline metal oxides, and the drying and thermal treatment of oxide films [23]. However, there has been comparatively less focus on studying chemical reactions in sol–gel solutions under microwave irradiation [24–27].

Numerous oxides have been synthesized by exposing precursor solutions to microwave irradiation, including MgO [28], RuO_2_ [29], ZrO_2_ [30], WO_3_ [31], SiO_2_ [32], TiO_2_ [33–34], and ZnO [12]. The microwave power utilized in these processes varied from 140 W [34] to 850 W [29]. ZnO is a widely studied n-type oxide semiconductor with many versatile and attractive applications in optical, optoelectronic, and photocatalytic fields [35–37]. The doping of ZnO with Mn can lead to the development of multifunctional nanostructures, such as room-temperature ferromagnetic materials with potential applications in spintronics and biological sensing [38–40]. The optical, thermal, and photocatalytic [40] reactivity of ZnO can also be improved with this method without changing its basic hexagonal structure. Consequently, the photodegradation of aqueous organic compounds triggered by manganese-doped ZnO still provides ample opportunities for future investigations despite the large number of such data already reported on ZnO materials.

Mn-doped ZnO materials synthesized by SG and its derivative methods can be successfully used for photodriven oxidation processes and water depollution since they are inexpensive, not toxic, and light-responsive. Up to now, advanced oxidation processes (AOPs) were used for the cleaning of waste water. Although AOPs based on engineered materials were performed in conjunction with biological treatments, the need for optimization still remains. Many photoactive semiconductors were proven to be efficient compounds for the photodegradation of organic pollutants from water, but this approach becomes really valuable when a green energy source (solar light) is used. Furthermore, photomineralization of aqueous pollutants is fully desirable because no harmful intermediates remain.

In previous studies, the authors demonstrated that Mn-doped ZnO films exhibit superior optical and piezoelectric properties compared to undoped ZnO, with a more compact microstructure and reduced surface roughness [41]. Building on this foundation, the current article aims to focus on the methods of preparation and provide a comparative analysis of Mn-doped ZnO powders synthesized using SG and MW. By doing so, the authors intend to further the understanding of how these preparation methods influence the properties and effectiveness of Mn-doped ZnO as a photocatalyst. The study emphasizes detailed morpho-structural characterizations of the resulting materials and investigates their photocatalytic performance for the mineralization of oxalic acid in aqueous solutions under simulated solar irradiation.

## Results and Discussion

### As-prepared samples

The synthesis process involved the preparation of precursor solutions comprising zinc acetate dihydrate, anhydrous manganese acetate, absolute ethanol, and triethanolamine. This was followed by gelation and subsequent drying and thermal treatment steps to yield Mn-doped ZnO nanopowders (see Experimental section). White-pink powders were obtained using both SG and MW methods.

#### Fourier-transform infrared spectroscopy

**Figure 1:**
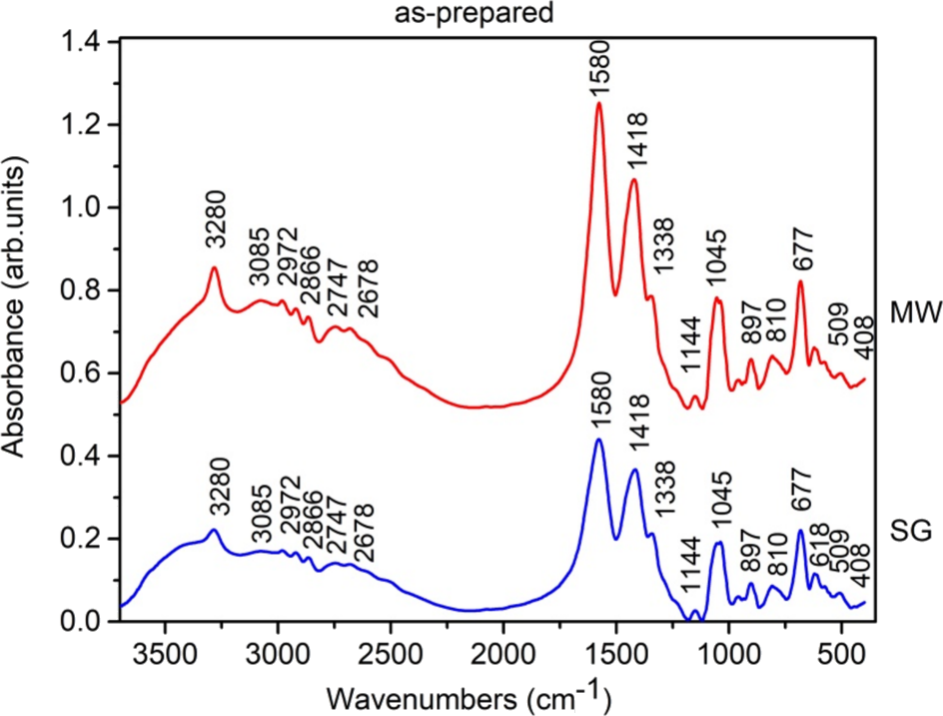
FTIR spectra of as-prepared gels.

Figure 1 shows the FTIR spectra of as-prepared gels obtained by SG and MW. The broad band in the 3600–2500 cm^−1^ region can be assigned to overlapping characteristic vibrations of C–H, N–H, and O–H bonds. The small bands at 2972, 2866, and 2747 cm^−1^ are characteristic to vibrations of C–H bonds in CH_2_ and CH_3_ groups.

The bands are shifted from their position in the spectrum of pure zinc acetate [42]. The asymmetric and symmetric stretchings of C=O in COO– groups occur in the FTIR spectra at 1580 and 1418 cm^−1^, respectively. The symmetric stretching of the C–O bond in COO– groups is present at 1045 cm^−1^ [42]. The small bands at 897 and 810 cm^−1^ are assigned to the stretching of C–C bonds and that at 509 cm^−1^ to the rocking of C–C bonds. The bands below 677 cm^−1^ are related to Zn–O vibrations [43–46]. The small bands at 408 and 421 cm^−1^ are characteristic of Mn–O bond vibrations [44]. Based on the FTIR spectra, we can assume that during the gelation process a zinc-based gel with a structure similar to the one reported by Moezzi [46] was obtained.

The primary distinction between the samples prepared by the two methods discussed in this article (SG and MW) lies in the intensity of the vibration bands, which is twice as high in the sample synthesized via MW. This difference can be attributed to the influence of microwave irradiation on the reaction rates within the sol–gel solutions, leading to enhanced vibrational band intensity.

#### Differential thermal analysis

**Figure 2:**
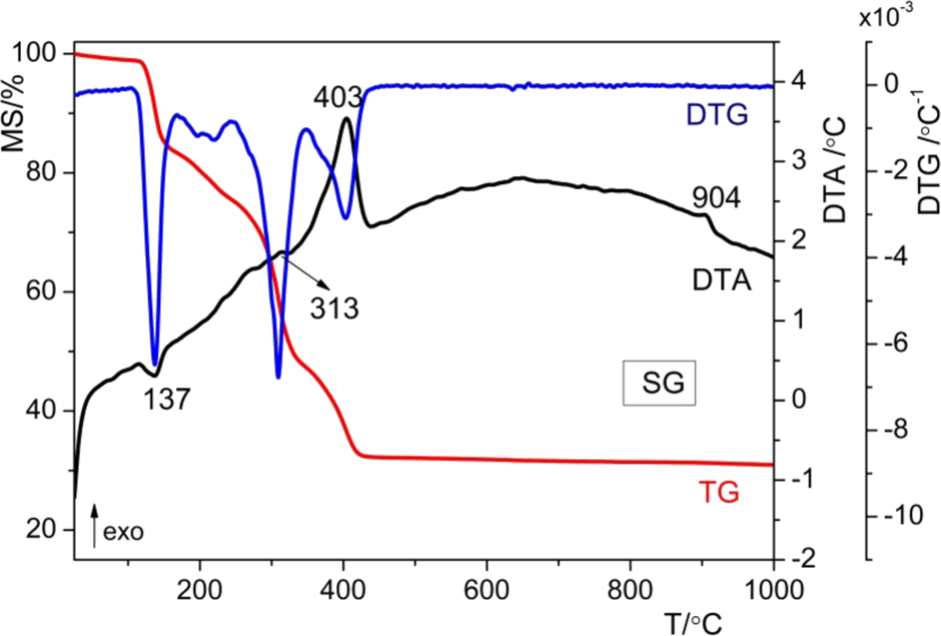
DTA, DTG and TG curves for the as-prepared SG powder.

**Figure 3:**
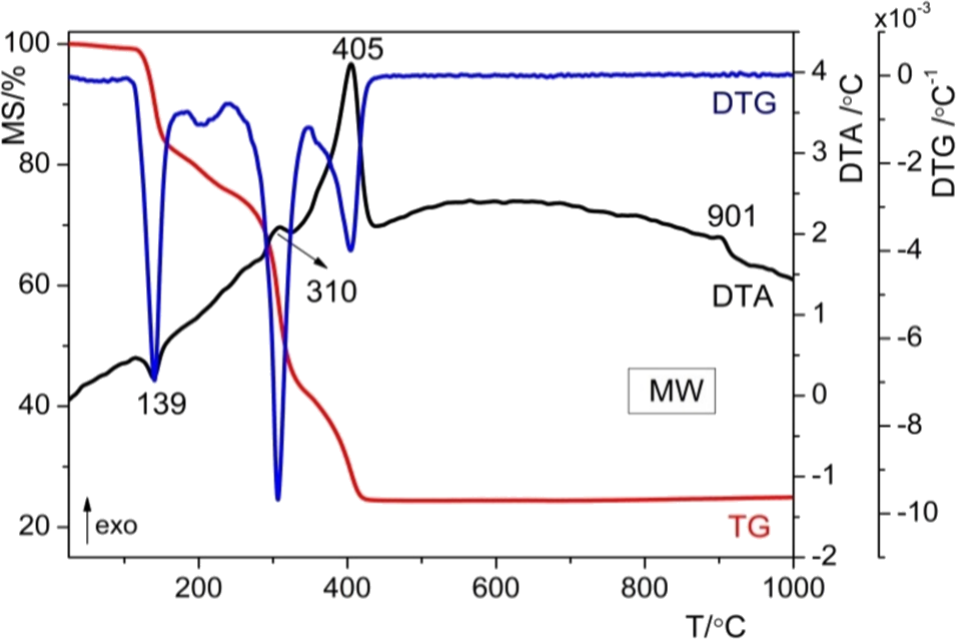
DTA, DTG and TG curves for the as-prepared MW powder.

The thermal behavior of the resulting materials was investigated by TG/DTG/DTA. It was shown that the thermal decomposition of the gels is not essentially influenced by the method of preparation. The thermo-gravimetric curves for the as-prepared Mn-doped ZnO powders are presented in Figure 2 and Figure 3. For the SG samples (Figure 2), the TG curve indicates a weight loss of approx. 69% up to 500 °C, while the DTA curve presents four effects, that is, an endothermic effect at 137 °C and three exothermic effects at 313, 403, and 904 °C. For the MW sample (Figure 3) the TG curve indicates a weight loss of approx. 75% up to 500 °C, while the DTA curve shows also four effects, that is, an endothermic effect at 139 °C and three exothermic effects at 310, 405 and 901 °C.

In both cases, the removal of physically absorbed water and solvent groups occurs between 100 and 180 °C, resulting in a mass loss of approximately 14.59% for the sol–gel (SG) sample and 17.07% for the microwave (MW) sample, accompanied by a minor endothermic peak around 140 °C. In the second phase, occurring between 180 and 350 °C for both samples, chemically bonded organic compounds are eliminated from the powders, leading to a mass loss of roughly 35% for SG and 40% for MW. This process is exothermic and observed around 309 °C. During the third phase, between 350 and 500 °C, structural hydroxy groups are removed, and any remaining organic material is burned off, resulting in a mass loss of approximately 11% and an exothermic effect around 405 °C. At higher temperatures, above 500 °C, there is one thermal effect noticed on the DTA curves, namely, an exothermal effect at 904 °C, which is assigned to ZnO crystallization. The decomposition ranges of the studied powders, the mass loses, and the corresponding thermal effects, as well as their assignments are summarized in Table 1. Microwaves intensify the sol–gel reactions but do not modify the formed molecular species, as has been shown for other oxide systems [14].

**Table 1:** TGA/DTA results for SG and MW samples.

Sample	Temperature range (°C)	Thermal effects (°C)		Mass loss (%)	Assignment

		endo	exo		

SG	25–100	—	—	1.30	physically absorbed water and solvent groups elimination
	100–180	139	—	14.59	decomposition elimination of organic species and structural hydroxy groups
	180–350	—	314	35.00	elimination of the structural hydroxy groups and burning out organic residues
	350–500	–—	404	15.73	elimination of the structural hydroxy groups and amorphous powder crystallization
	500–1000	—	905	2.60	crystallization
				sum = 69.22	

MW	25–100	—	—	0.72	physically absorbed water and solvent groups elimination
	100–180	140	—	17.07	decomposition and elimination of organic species
	180–350	—	308	40.00	burning out organic residues
	350–500	—	405	17.85	elimination of the structural hydroxy groups and transformation from amorphous to crystalline powder
	500–1000	—	904	0.55	crystallization
				sum = 76.19	

#### Thermally treated samples

Based on the results obtained by thermal analysis, the as-prepared samples were thermally treated at 350 °C and 500 °C for 1 h each.

#### Scanning electron microscopy

**Figure 4:**
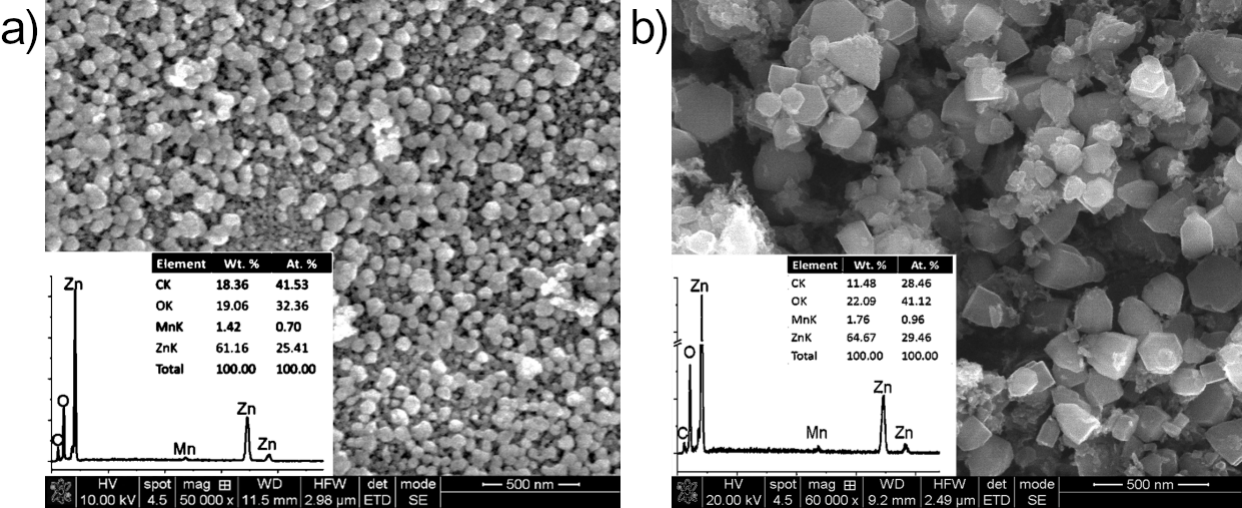
SEM images for (a) SG sample and (b) MW sample.

The morphology and chemical composition (inset) of the thermally treated samples is illustrated in Figure 4. Homogeneously distributed, quasi-spherical nanoparticles (mean size diameter of 70 nm) are observed in the SG sample (Figure 4a), while larger, well-defined prismatic particles are found in the MW sample. EDX analysis highlights the presence of Mn in both samples.

#### Fourier-transform infrared spectroscopy

**Figure 5:**
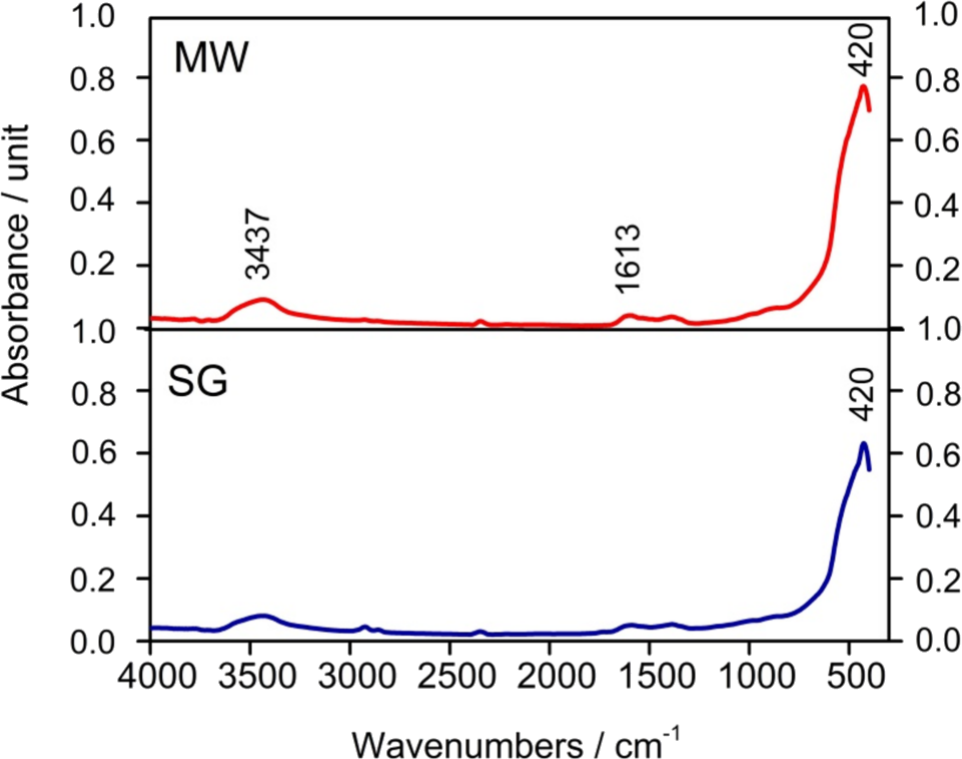
FTIR spectra of the thermally treated samples (SG and MW).

Figure 5 presents the FTIR spectra of the thermally treated samples, revealing the absence of bands characteristic of organic groups after thermal treatment. Notably, the main vibration band of Zn–O–Mn is observed at 420 cm^−1^, as reported in previous studies [23]. The higher intensity of this band in the nanopowder synthesized via MW is evident. This increased band intensity at 420 cm^−1^ suggests enhanced crystallization, consistent with the results from XRD and SEM analyses. Additionally, bands at 3437 and 1613 cm^−1^ correspond to the vibrational modes of hydroxy groups (OH) bonded to the surface of the ZnO powders.

#### X-ray diffraction

**Figure 6:**
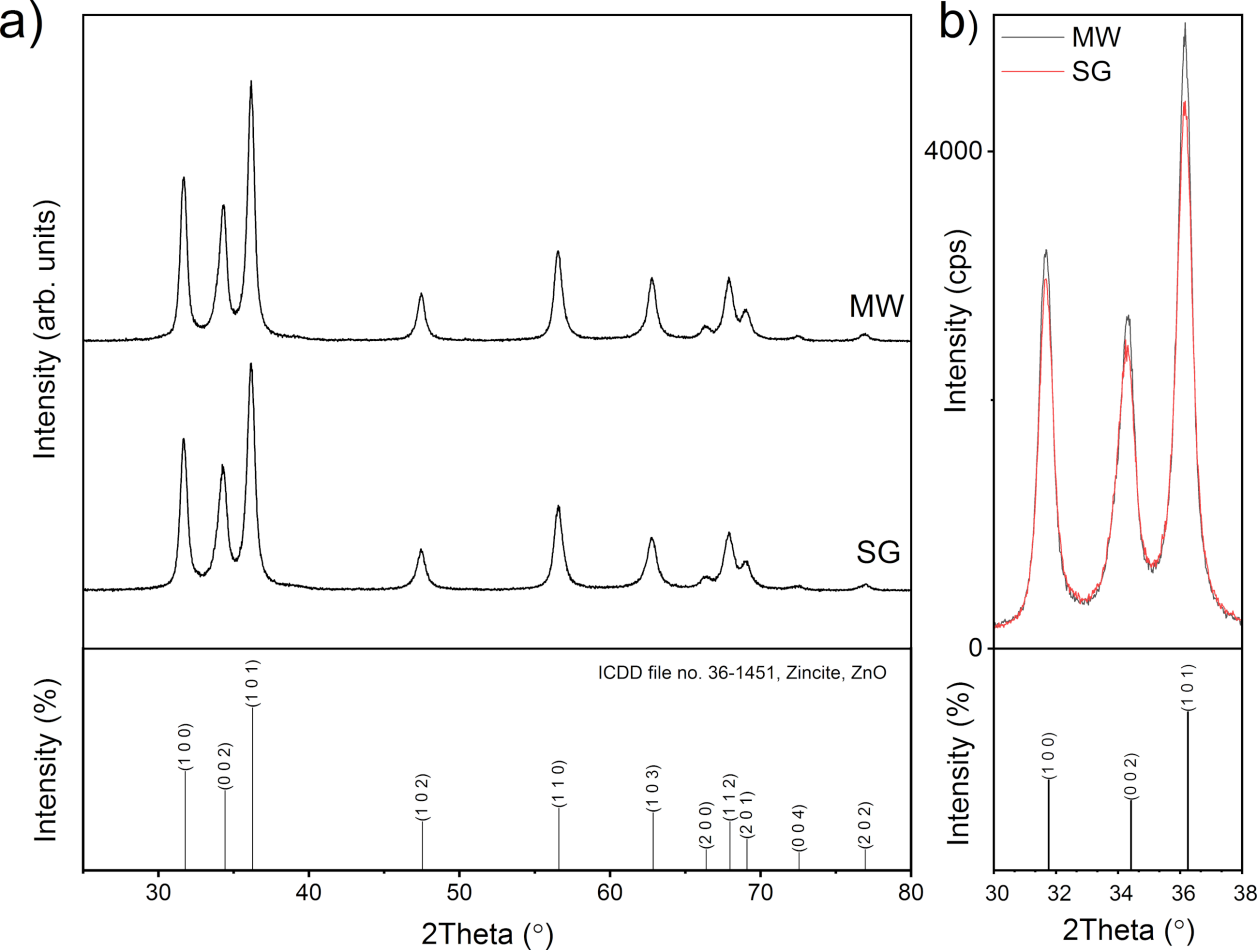
XRD patterns of SG and MW powders, thermally treated at 500 °C, over (a) the full 2θ range and (b) a selected 2θ range for intensity comparison.

The crystalline structure of the samples thermally treated at 500 °C was analyzed using X-ray diffraction (XRD). The resulting XRD patterns, shown in Figure 6a, indicate that the samples are polycrystalline and single-phase. This single phase corresponds well to the data from ICDD file no. 36-1451 of zincite ZnO. ZnO has a wurtzite-type structure and crystallizes in the hexagonal *P*6_3_/*mc* space group. No other phases, including Mn-based compounds, were detected within the detection limits of the instrument, suggesting that the dopant has been incorporated into the zincite lattice. The patterns of the two samples, SG and MW, are very similar, with slightly more intense diffraction lines for the MW sample. This difference in intensities, as it can be noticed in Figure 6b, can be attributed to the microwave irradiation of the sol–gel solution (sample MW) [47].

Table 2 presents the lattice parameters, average crystallite sizes, and lattice strains. The average crystallite sizes and lattice strains were determined using the Halder–Wagner method [48] based on the integral line width. By plotting these values, the crystallite size (*L*) can be derived from the slope of the approximation line, while the lattice strain (ε) is obtained from the intercept of the y axis.

**Table 2:** Lattice parameters, mean crystalline domain sizes, and lattice strains.

Sample	Lattice constants		Crystallite size *L* (Å)	Lattice strain ε (%)

	*a* (Å)	*c* (Å)		

SG	3.2507(18)	5.206(4)	135.04(19)	0.0051(5)

MW	3.2516(14)	5.216(3)	147.9(2)	0.0042(6)

ZnO (ICDD file 26-1451)	3.2500	5.207	—	—

The lattice constants, listed in the Table 2, are very close to the values of ICDD file no. 36-1451. The mean crystallite size of the MW sample is 1 nm larger than that of sample SG. The lattice strain follows an inverse trend, and the value of the strain in the SG sample is larger than the value of the sample MW. This can be explained by the influence of the microwave irradiation, which is known to create materials with fewer defects [49].

#### Porosimetry

**Figure 7:**
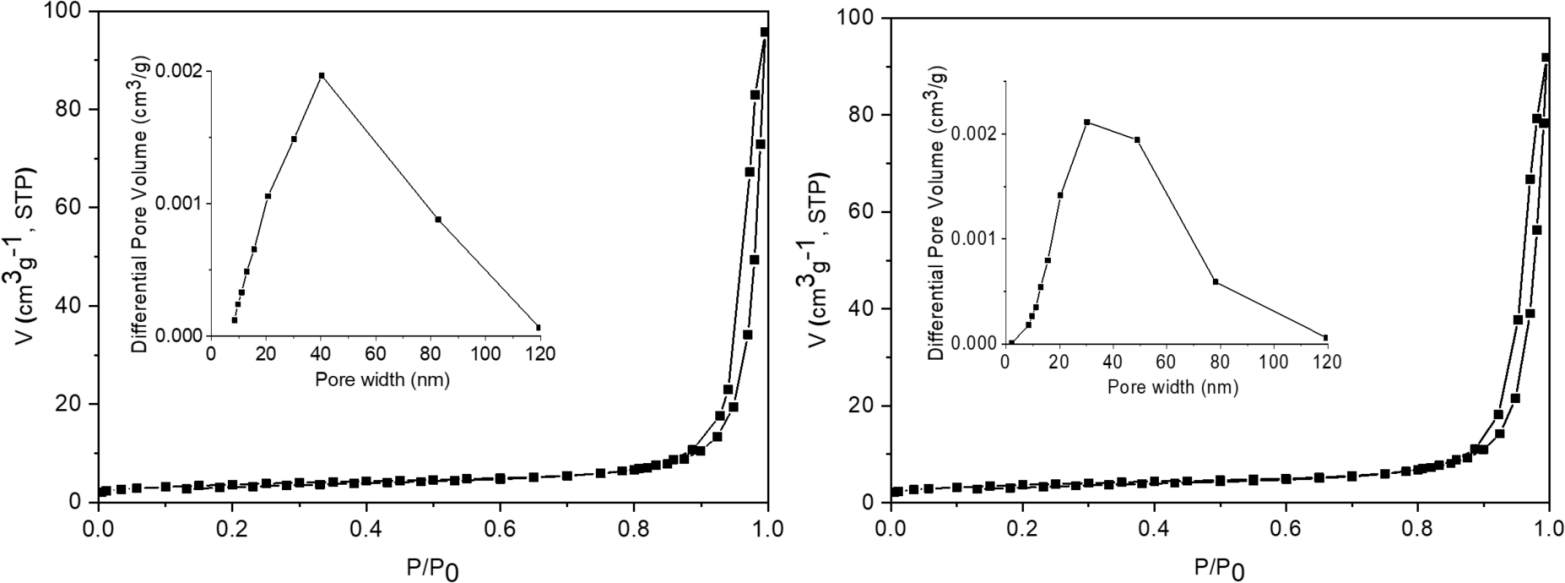
N_2_ adsorption–desorption isotherms and pore size distributions (inset of the figures) for the investigated samples: SG (left) and MW (right).

The nitrogen adsorption–desorption isotherms and pore size distributions are presented in Figure 7. The adsorption and desorption branches for both samples appear to be almost parallel in Figure 7. The insets show a wide pore size distribution reaching 120 nm and pore width maxima located in the mesoporosity area for both samples (40 nm for SG and 35–45 nm for MW). Similar textural features for SG and MW samples are presented in Table 3.

**Table 3:** Textural parameters of the samples.

Sample	*S*_BET_ (m^2^·g^−1^)	*V*_total_ (cm^3^·g^−1^)	Average pore diameter (nm)

SG	12.6	0.148	41.9
MW	12.8	0.142	40.1

#### UV–vis spectroscopy

**Figure 8:**
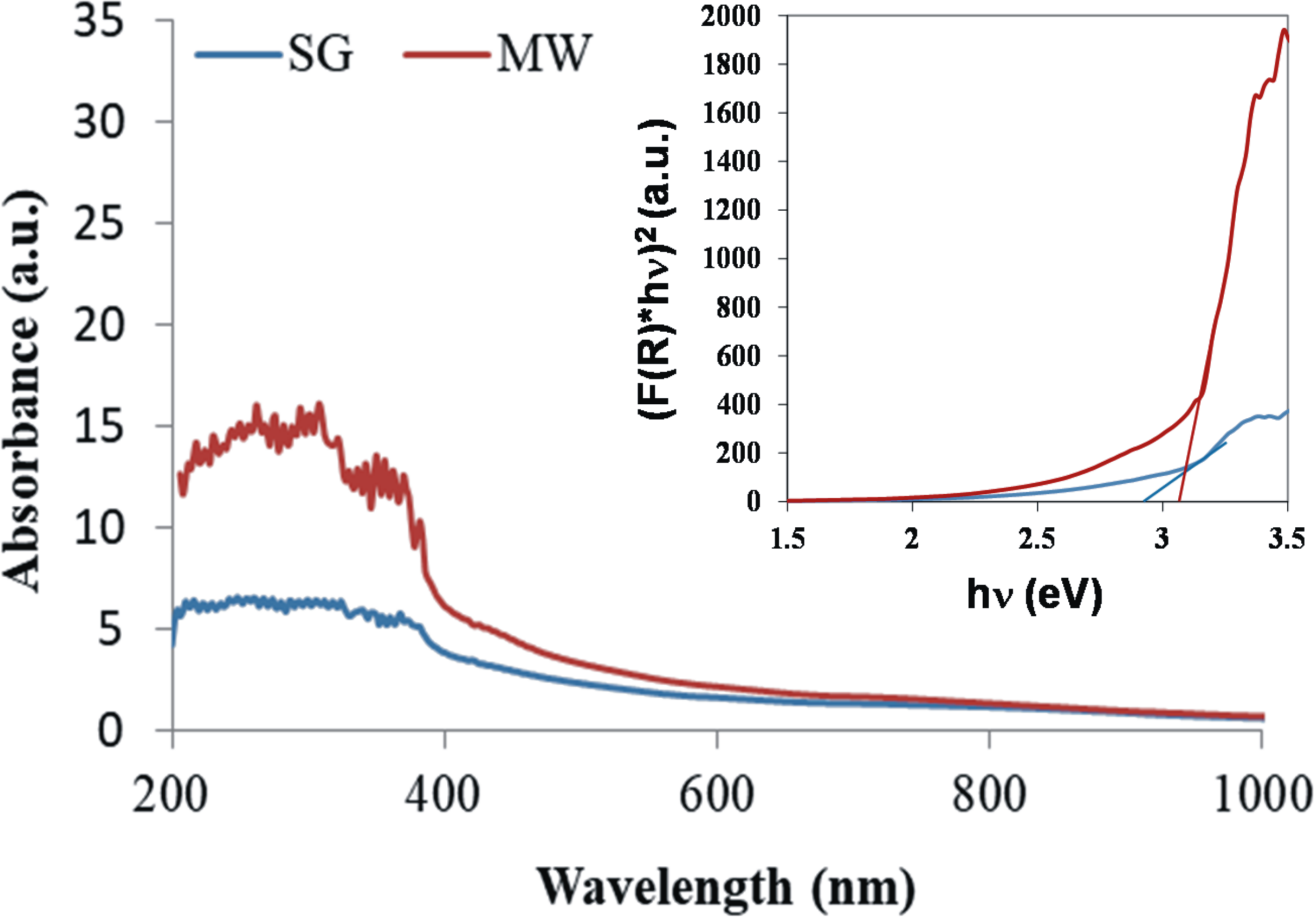
Comparative UV–vis spectra of SG and MW; the inset illustrates the Tauc plot for SG and MW samples.

The recorded UV–vis spectra (Figure 8) depict the light absorption mainly in the 200–400 nm range for both samples. The MW sample exhibits a higher light absorption and a tendency of main broad band to split at 330 nm. The long absorption tails in the visible domain can be assigned to the presence of surface defects [50].

The direct transitions represented in the inset allow one to evaluate the forbidden band energies, *E*_g_(SG) = 2.95 eV and *E*_g_(MW) = 3.05 eV, indicating a slighty higher photoreactivity of the SG sample. Supporting Information File 1, Figure S2 presents comparative UV–vis spectra for the undoped SG ZnO and MW ZnO samples, with larger *E*_g_ values than those of the manganese-doped samples.

#### Photoluminescence

**Figure 9:**
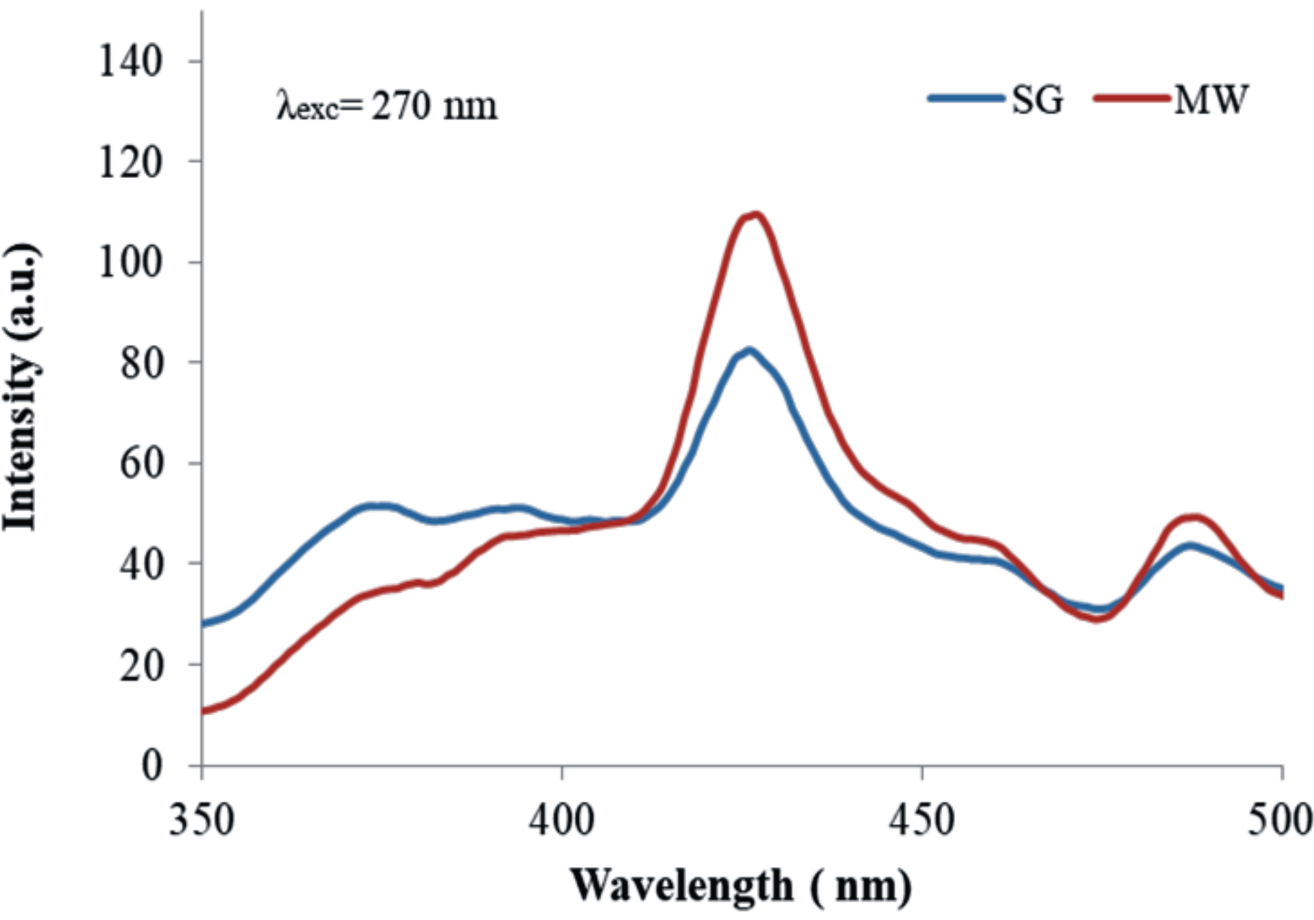
Photoluminescence spectra of the powders suspended in water (SG and MW) collected for λ_exc_ = 270 nm.

Photoluminescence (PL) measurements are usually used to describe the radiative recombinations of electron–hole pairs in semiconductors exposed to light irradiation. A high PL signal measured for a catalyst is generally associated with low photocatalytic activity. Accordingly, various modifiers of semiconductor nanomaterials are used to enhance separation of the photogenerated charges, causing a corresponding decrease of PL emission. The correlation between photoluminescence and photocatalytic activity of the modified nanomaterials can be modulated by the dopant concentration. Figure 9 shows for both investigated samples a main emission peak at 420 nm and a smaller one at 480 nm. According to the literature data, both maxima correspond to the excitonic PL related to surface oxygen vacancies and defects of ZnO nanoparticles. The first peak is attributed to band edge free excitons [51] and the second to bound excitons [51–52].

**Figure 10:**
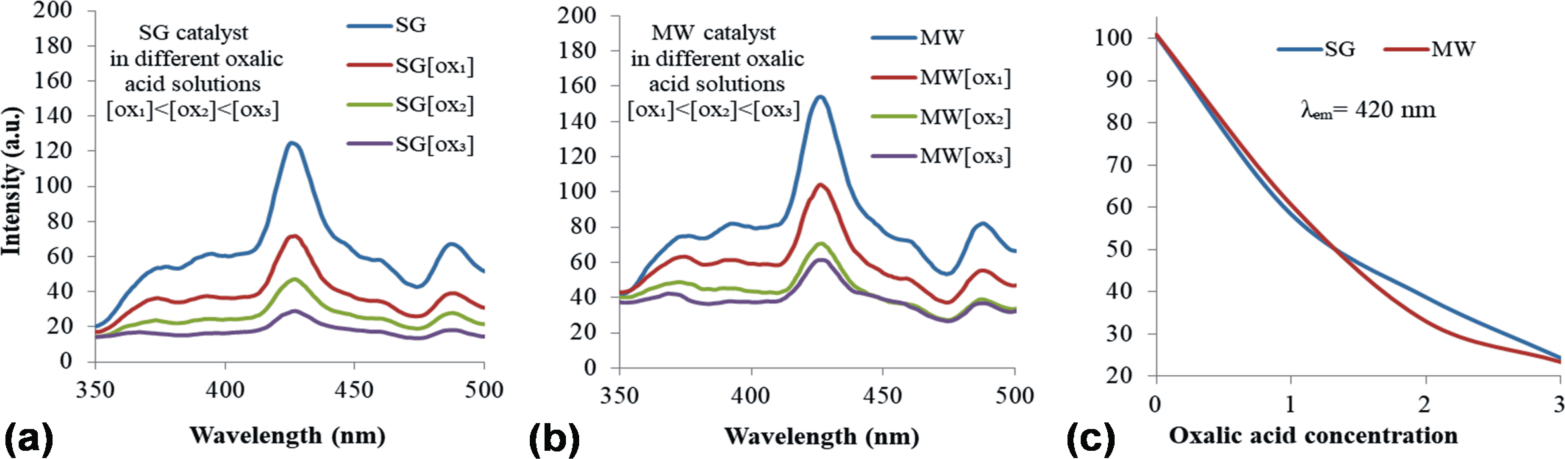
Quenching of catalyst photoluminescence with increasing oxalic acid concentrations in aqueous solutions: (a) SG catalyst, (b) MW catalyst; (c) decreasing of emission signal at 420 nm with increasing oxalic acid concentrations.

The lower PL signal of the SG sample relative to the MW sample suggests a slower rate of recombination for photogenerated charges and, consequently, a much more efficient use in photocatalysis processes (i.e., degradation of oxalic acid). In addition, the measured excitonic PL indicates the need for deeper investigation on the ability of the investigated materials to generate reactive oxygen species (ROS) under light irradiation. The photoluminescence signal of the powders suspended in aqueous oxalic acid solutions depends on the oxalic acid concentration as shown in Figure 10.

The progressive PL quenching with increasing oxalic acid concentration is a clear proof for the consumption of photogenerated charges by oxalic acid adsorbed on the surface. Briefly, Figure 10 reveals two significant aspects: (i) The main peak emission for both powders suspended in oxalic acid solutions diminishes. This may be suggestive for the charge carrier type (electrons or holes) acting in the photo-mediated interaction of the surface catalyst with the reactant [53]. (ii) The SG sample tends to a complete quenching of the photoluminescence signal with increasing concentrations, while the MW sample exhibits a plateau (Figure 10c). The latter observation can be associated with the adsorption degree of oxalate ions on the catalyst surface.

The two abovementioned observations indicate a small difference of catalyst activity in the reaction medium (exposure to solar irradiation and presence of oxalic acid/degradative products), indicating structural differences that are worthy to be deeply investigated in a future dedicated study.

#### Generation of reactive oxygen species

**Figure 11:**
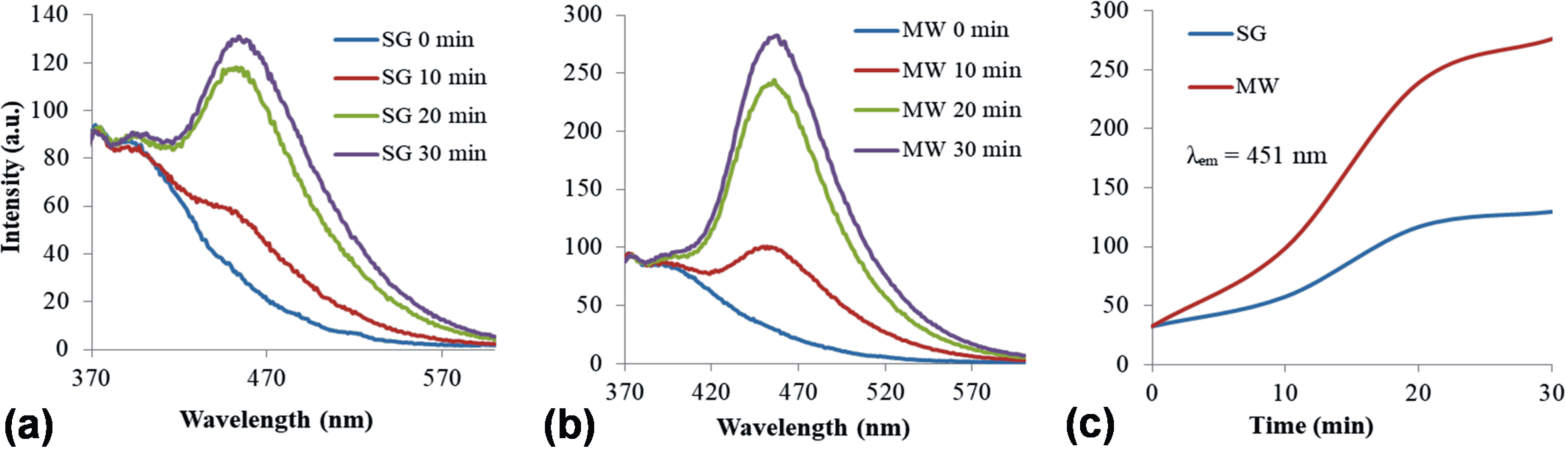
Generation of hydroxyl radicals by (a) SG and (b) MW (b) catalysts. (c) Comparative emissions at 451 nm assigned to umbelliferone formation under simulated solar irradiation.

ROS are usually involved in the photodegradation of organic compounds in aqueous media as previously reported [54]. The generation of hydroxyl radicals (·OH) by SG and MW catalysts under simulated solar light irradiation (AM1.5) was determined from the PL emission at 451 nm assigned to the presence of a fluorescent compound, namely umbelliferone, a coumarin degradation product obtained in the presence of hydroxyl radicals. Figure 11a–c clearly shows that ·OH radicals are photogenerated to a greater extent by the MW catalyst. This could be the result of specific differences in surface chemistry of the two investigated samples.

**Figure 12:**
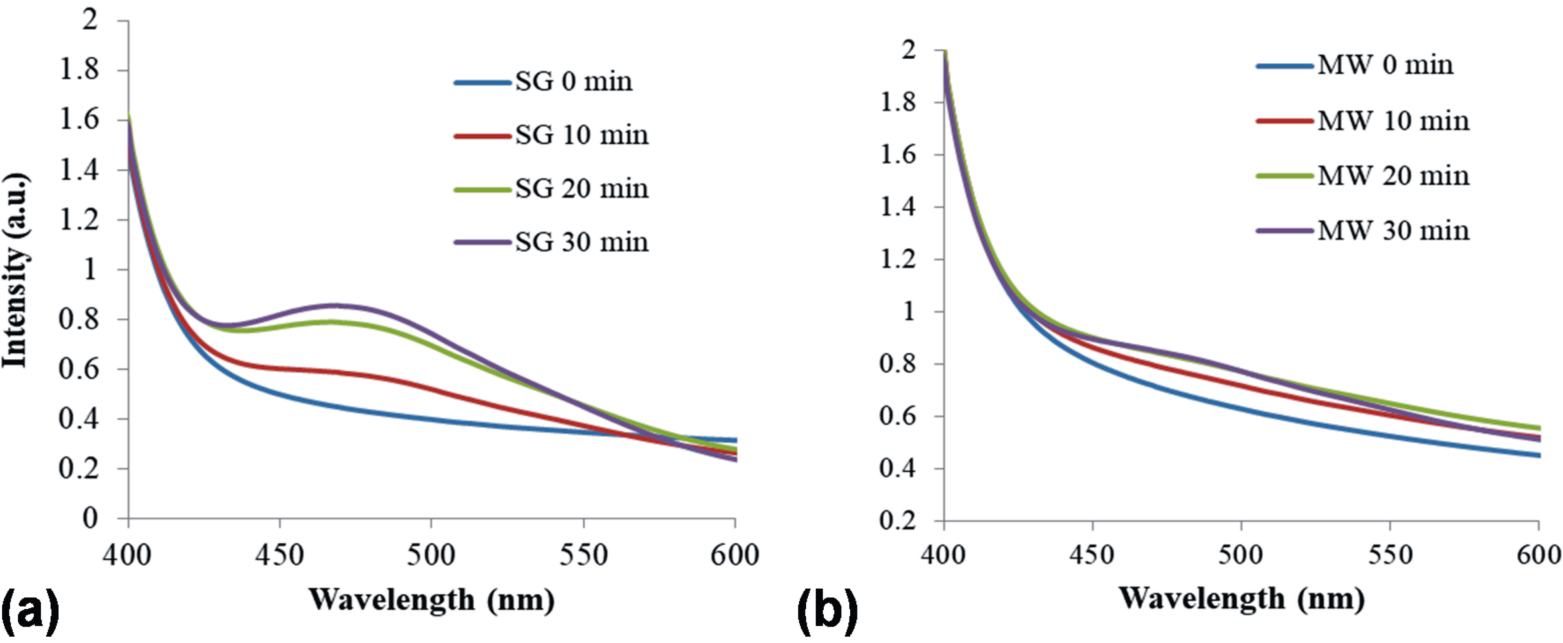
O_2_^−^ formation over SG and MW powders under simulated solar irradiation.

The ability of SG and MW catalysts to generate superoxide anions (O_2_^−^) under simulated solar light irradiation is shown in Figure 12. This was spectroscopically monitored taking into account the intensity of the characteristic peak of formazan (485 nm). This compound results from the reaction of 2,3-bis(2-methoxy-4-nitro-5-sulfophenyl)-2*H*-tetrazolium-5-carboxanilide (XTT sodium salt) and the photogenerated O_2_^−^. An obvious difference appears in Figure 12 regarding the higher ability of the SG sample to produce O_2_^−^ relative to MW. This observation can be correlated with the lower PL signal of the SG sample (assigned to surface oxygen vacancies and defects) and can be further analyzed taking into account the photocatalytic test results.

#### Photocatalytic activity

**Figure 13:**
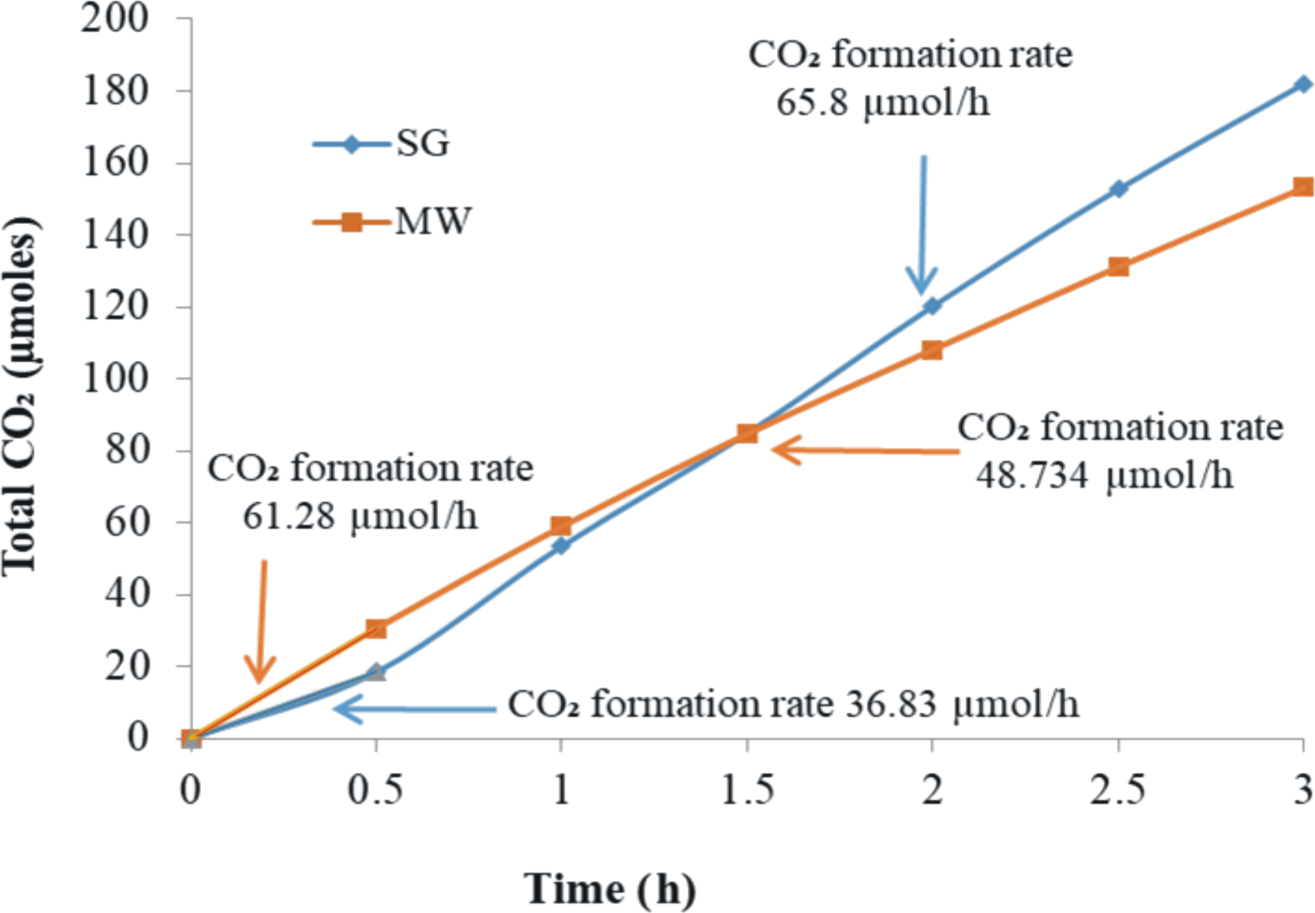
Photoactivity of the suspended catalyst powders for oxalic acid oxidation to CO_2_ under simulated solar light irradiation (AM1.5).

Oxidative photodegradation of oxalic acid in aqueous solution (Figure 13) was used to compare the catalytic activity of the materials obtained by SG and MW. The overall photocatalytic response of each catalyst was evaluated taking into account the blank experiment (oxalic acid photodegradation test carried out in the absence of catalyst). The CO_2_ generation was monitored in the aqueous phase under simulated solar irradiation for 3 h. The obtained amounts were 182 µmol of CO_2_ for the SG catalyst and 153 µmol of CO_2_ for the MW catalyst. However, Figure 13 depicts particular aspects concerning the CO_2_ generation for each catalyst in the time course of the photocatalytic process. In the case of SG, the CO_2_ formation rate is lower at the beginning (36.83 µmol·h^−1^) and increases significantly in the following 2.5 h (65.8 µmol·h^−1^). Unlike this, the MW catalyst appears to be much more active in the first half hour (61.28 µmol·h^−1^) than later (48.73 µmol·h^−1^). H_2_ production was also checked but only traces were found for both catalysts. This observation can be correlated to the ability of the SG material to produce O_2_^−^ by electron trapping. In contrast, the ·OH generation in the presence of the MW catalyst could explain the higher formation rate of CO_2_ from the beginning of the photocatalytic process because of the high oxidation capacity of hydroxyl radicals. The subsequent decrease of the CO_2_ formation rate could be assigned to a presumed catalyst deactivation due to the strong adsorption of reactant/reaction products on its surface. This observation is in line with the PL measurements in oxalic acid solutions and the FTIR post-reaction spectra (Supporting Information File 1, Figure S1). Zeta potential measurements revealed values of +17.48 mV and +11.09 mV for SG and MW samples, respectively, suggesting a better adsorption of oxalate ions on the SG sample than on the MW sample.

The ability of the photocatalysts to be reused has been checked over the course of three reaction cycles, in which the SG sample proved to be more stable. The amount of evolved CO_2_ decreased from the first to the third reaction cycle by 10% for SG compared to 15% for MW.

Briefly, based on the previously presented data, a significant role of the ROS in photomineralization of oxalic acid over the two investigated catalysts can be assumed. The authors’ previous photocatalytic studies concerning the photodegradation of oxalic acid emphasized the dependence of photocatalytic activity on catalyst morphology [55]. The importance of the catalyst bandgap and its surface chemistry relative to reactant adsorption and photocatalytic activity were also clearly shown [56]. According to the abovementioned data, the possible routes for the present photocatalytic process can be described as follows:


[1] $$\text{Cat.}+h\nu \to {\text{e}}^{-}+{\text{h}}^{+}$$

[2] $${\text{H}}_{2}{\text{C}}_{2}{\text{O}}_{4}\to {\text{C}}_{2}{\text{O}}_{4}^{\;2-}+2{\text{H}}^{+}$$

[3] $$2{\text{h}}^{+}+{\text{C}}_{2}{\text{O}}_{4}^{\;2-}\to 2{\text{CO}}_{2}$$

[4] $${\text{h}}^{+}+\;-\text{ OH (surface hydroxy)}\to \cdot \text{OH}$$

[5] $${\text{O}}_{2}+{\text{e}}^{-}\to {\left({\text{O}}_{2}^{\;-}\right)}_{\text{ads}}\;(\text{on catalyst surface})$$

[6] $${\left({\text{O}}_{2}^{\;-}\right)}_{\text{ads}}+2{\text{H}}^{+}+{\text{e}}^{-}\to {\text{H}}_{2}{\text{O}}_{2}\to \text{2}\cdot \text{ OH}$$

[7] $${\text{C}}_{2}{\text{O}}_{4}^{\;2-}+2\cdot \text{ OH}\to 2{\text{CO}}_{2}+{\text{2OH}}^{-}$$

[8] $$2{\text{H}}^{+}+2{\text{e}}^{-}\to {\text{H}}_{2}$$

The main question to be answered is which of them is responsible to a greater extent for the particular photocatalytic behavior of the investigated materials. The overall results regarding the amount of evolved CO_2_ for both samples indicate the importance of Equation 3. Equation 4 suggests a competing hole consumption also indicated by ROS investigations. In the case of the MW sample, it is presumable that the photogenerated holes are mainly consumed through the formation of hydroxyl radicals, which reduces the amount of available h^+^ for the direct oxidation of C_2_O_4_^2−^ (Equation 3). Further investigations are needed for a complete evaluation of the photocatalytic properties of SG and MW. Based on these preliminary results, it is fully desirable to develop such engineered materials [57] for the removal of organic pollutants. It is important to expand the activity range of the materials from UV to visible light by tailoring the manganese addition to ZnO and to examine the photocatalytic mechanism under different irradiation sources. Also by the sol–gel method, the active materials can be deposited in future studies as films on different supports for better surface contact and recovery degree, enhancing the scale-up opportunity for water cleaning technologies.

Despite the many works dealing with ZnO-based materials dedicated to catalytic applications, there is still room for investigation on inexpensive, noble metal-free active materials under solar irradiation. These materials are more valuable when targeting the organic pollution mineralization from waters.

## Conclusion

This study presents a comparative analysis of Mn-doped ZnO nanopowders synthesized using both sol–gel and microwave-assisted sol–gel methods. The structural, morphological, and optical characteristics of the resulting powders were comprehensively evaluated, demonstrating that both synthesis techniques are effective in producing photocatalytically active materials based on manganese-doped ZnO. Despite the similar start composition, obvious morphological differences, structural defects, and surface chemistry particularities were revealed for the investigated samples, which affected the photogeneration of reactive oxygen species.

The photocatalytic assays clearly showed the ability of both investigated catalysts to trigger the mineralization of oxalic acid under solar irradiation, where the total amount of evolved CO_2_ was slightly higher for the SG sample. Still, using sol–gel synthesis assisted by microwave irradiation has the advantage of saving time and energy.

Developing new depollution technologies to mineralize organic compounds in aqueous media under solar light irradiation is now mandatory for environment preservation. The above-investigated materials proved to fit perfectly well to these requirements, putting in front inexpensive, environmentally friendly ZnO able to remove the oxalic acid from aqueous media under solar light irradiation, without added noble metals and only modified with manganese.

Our findings not only highlight the potential of Mn-doped ZnO as a highly effective photocatalyst for organic pollutant degradation but also underscore the importance of optimizing synthesis methods to enhance efficiency and reduce energy consumption. These advancements are crucial for addressing pressing environmental challenges and pave the way for more sustainable approaches to water purification and pollutant remediation.

## Experimental

### Synthesis

The starting materials (all reagent grade) for obtaining precursor solutions are zinc acetate dehydrate (Zn(CH_3_COO)_2_·2H_2_O), anhydrous manganese acetate (Mn(CH_3_COO)_2_), absolute ethanol (C_2_H_5_OH), and triethanolamine (C_6_H_15_NO_3_, TEA). The precursor solutions 0.1 M of (0.098% Zn^2+^ + 0.002% Mn^2+^) were stirred in absolute ethanol at 50 °C for 15 min. Then, triethanolamine in a molar ratio of ZnAc/TEA = 5:1 was slowly added drop wise. The resulted solution was homogenized at 50 °C for 2 h in the sol–gel method without microwaves or exposed to microwaves for 10 min at 60 °C, 200 W power and a frequency of 2.45 GHz. Gelation took place at room temperature. All resulting gels were dried at 100 °C for 24 h and then thermally treated at 350 °C for 1 h with a heating speed of 1 °C/min followed by a treatment at 500 °C for 1 h with a heating speed of 5 °C/min.

### Characterization

Fourier transform infrared spectra (FTIR) were recorded with a Nicolet 6700 apparatus in the range of 400–4000 cm^−1^. The resolution was 4 cm^−1^. The powders were pressed in KBr pellets, and the spectra were recorded in absorbance mode.

Differential thermal analysis (DTA) and thermogravimetric analysis (TGA) were used to determine the thermal behavior of the synthetized powders by using a Mettler Toledo TGA/DTA 851e apparatus with Al_2_O_3_ crucibles and flowing air atmosphere at 80 mL/min. The maximum temperature was set at 1000 °C and the heating rate was 5 °C/min.

The surface morphologies and the compositions of the calcined samples were evaluated by scanning electron microscopy (SEM-EDS), using a FEI Quanta 3D FEG instrument equipped with the Octane Elect EDS system. The powder samples were placed on double-sided carbon tape and recorded without coating at accelerating voltages of 10 and 20 kV in high vacuum mode.

X-ray diffraction (XRD) measurements were carried out using an Ultima IV diffractometer (Rigaku Corp., Japan) equipped with parallel beam optics, using Cu Kα radiation (λ = 1.5418 Å) at 40 kV and 30 mA over the 2θ range of 25–80° at a scanning rate of 2°/min with a step width of 0.02°. The phase identification was performed using the search/match algorithm connected to (ICDD PDF-2).

Nitrogen adsorption–desorption isotherms (BET) at 77 K were recorded on a Micromeritics ASAP 2020 analyzer (Norcross, GA, USA). The samples were degassed at 200 °C for 4 h under vacuum before analysis. Specific surface areas (S-BET) were calculated according to the Brunauer–Emmett–Teller (BET) equation, using adsorption data in the relative pressure range between 0.05 and 0.30. The pore size distribution curves were obtained from the desorption data using the BJH (Barrett–Joyner–Halenda) model.

Diffuse reflectance UV–vis spectra were recorded with a Perkin Elmer Lambda 35 spectrophotometer equipped with an integrating sphere. The analysis range was 200–1000 nm. Based on the Kubelka–Munk function, the collected reflectance data were transformed into absorption spectra. *E*_g_ was determined using the Tauc plot method, considering direct transitions. The method consists in the extrapolation of the linear region of the rising part of the curve (α*h*ν)^1/η^ as a function of *h*ν to zero to get the bandgap (where α is the Kubelka–Munk function, *h*ν is the energy of the photons, and η has the value 1/2 for direct-bandgap semiconductors and 2 for indirect-bandgap semiconductors or amorphous compounds.

Photoluminescence measurements (PL) were carried out using a Carry Eclipse fluorescence spectrometer from Agilent Technologies and the following parameters: scan rate of 120 nm·min^−1^, spectral resolution of 0.5 nm, and 10 nm slits in excitation and emission. All measurements were performed for 0.001 g catalyst suspended after ultrasonication in ultrapure water at room temperature (25 °C) and λ_exc_ = 270 nm.

For ROS identification, ·OH radicals trapping was performed with 10 mM coumarin solution after simulated solar irradiation of 0.001 g suspended catalyst leading to the formation of fluorescent umbelliferone. This was monitored with a Carry Eclipse fluorescence spectrometer, slits set to 10 nm in excitation and emission, λ_exc_ = 330 nm.

O_2_‾ formation was measured by exposing the catalyst to simulated solar light, as follows: 0.003 g powder was suspended in 3 mM solution of 2,3-bis(2-methoxy-4-nitro-5-sulfophenyl)-2*H*-tetrazolium-5-carboxanilide; its reduction by the photogenerated O_2_‾ leads to the formation of formazan. This is signaled by a broad peak located at 470 nm. The UV–vis spectra were recorded with an Analytik Jena Specord 200 Plus spectrophotometer.

Photocatalytic tests were performed in a photoreactor provided with a quartz window and thermostated at 180 °C by a chiller. A quantity of 0.025 g catalyst was suspended by sonication in 120 mL aqueous solution containing 0.1 g oxalic acid. AM1.5 light provided by a solar simulator (Peccell-L01) equipped with a xenon short-arc lamp (150 W) was used for the photocatalytic tests. Ar (carrier gas, 10 cm^3^/min) together with oxygen (1 cm^3^/min) was bubbled into the oxalic acid solution. The gaseous products of interest, H_2_ and CO_2_, were investigated every 30 min by a gas chromatograph (Buck Scientific) equipped with molecular sieve 5 Å and Haysept columns. The photocatalytic experiments were triplicates, and the represented data are the mean values.

Zeta potential measurements were conducted using a Beckman Coulter Delsa Nano C analyzer (Brea, CA, USA) at 25 °C. The samples were prepared in ultrapure water at a concentration of 1.25 mg/mL.

## Supporting Information

Figure S1 displays the FTIR spectra of the sol–gel (SG) and microwave-assisted sol–gel (MW) samples after photocatalytic tests, alongside the spectrum of commercial oxalic acid for comparison. Figure S2 shows the UV–vis spectra of pristine sol–gel ZnO and microwave ZnO samples.

File 1 Additional experimental data.

## Data Availability

All data that supports the findings of this study is available in the published article and/or the supporting information to this article.
