# Supplementary material for: Mn-doped ZnO nanopowders prepared by sol–gel and microwave-assisted sol–gel methods and their photocatalytic properties
Source: Beilstein J Nanotechnol. 2024 Oct 28;15:1283–96. doi: 10.3762/bjnano.15.104 (PMC11535566; doi:10.3762/bjnano.15.104)
Supplement: File 1 — Additional experimental data. [file Beilstein_J_Nanotechnol-15-1283-s001.pdf]

BEILSTEIN JOURNAL OF NANOTECHNOLOGY

Supporting Information

for

Mn-doped ZnO nanopowders prepared by sol–gel and microwave-assisted sol–gel methods and their photocatalytic properties

Cristina Maria Vlădu[redacted], Crina Anastasescu, Silviu Preda, Oana Catalina Mocioiu, Simona Petrescu, Jeanina Pandele-Cusu, Dana Culita, Veronica Bratan, Ioan Balint and Maria Zaharescu

Beilstein J. Nanotechnol. **2024,** *15,* 1283–1296. doi:10.3762/bjnano.15.104

Additional experimental data

License and Terms: This is a supporting information file under the terms of the Creative Commons Attribution License (https://creativecommons.org/licenses/by/4.0). Please note that the reuse, redistribution and reproduction in particular requires that the author(s) and source are credited and that individual graphics may be subject to special legal provisions.
The license is subject to the *Beilstein Journal of Nanotechnology* terms and conditions: (https://www.beilstein-journals.org/bjnano/terms)

Figure S1 displays the FTIR spectra of the sol–gel (SG) and microwave-assisted sol–gel (MW) samples after photocatalytic tests, alongside the spectrum of commercial oxalic acid for comparison. The spectra reveal the adsorption of oxalic acid on the surface of both SG and MW samples. The positions of the oxalic acid bands are shifted, indicating a chemical interaction with the zinc oxide.

In commercial oxalic acid, the asymmetric and symmetric stretching of the C=O bond in the COOH groups appear at 1692 cm^{-1} and 1443 cm^{-1}, respectively. However, in the spectra of the SG and MW samples, only the band corresponding to the asymmetric stretching of C=O is present at 1647 cm^{-1}, shifted to a lower wavenumber. The asymmetric and symmetric stretching of the C–O bond in the COOH groups, originally at 1250 cm^{-1} and 1132 cm^{-1} in commercial oxalic acid, are shifted to 1363 cm^{-1} and 1318 cm^{-1}, respectively, in the SG and MW samples. This shift is attributed to the adsorption of oxalic acid on the Mn-doped ZnO surfaces through hydrogen bonding.

In the commercial oxalic acid spectrum, the O-H stretching bands appear at 3525 cm^{-1} and 3433 cm^{-1}, whereas the SG and MW sample spectra exhibit a single, asymmetric band at 3383 cm^{-1}. This indicates a change in the structure. The observed spectral shifts of the OH-stretching vibrational transitions were recorded at 142 cm^{-1} for both SG and MW samples.

In the spectrum of oxalic acid, small bands corresponding to the scissoring, rocking, and twisting vibrations of the C–C and C–OH bonds are observed below 722 cm^{-1}. In the spectra of the SG and MW samples, these bands are shifted to higher wavenumbers, and their number increases, suggesting alterations in the molecular interactions.

In conclusion, after the photocatalytic tests, oxalic acid was detected in the spectra of both Mn-doped ZnO samples. It is important to note that the FTIR analysis in this study is qualitative and does not provide information on the quantity of acid deposited on the surfaces of the SG and MW samples separately.

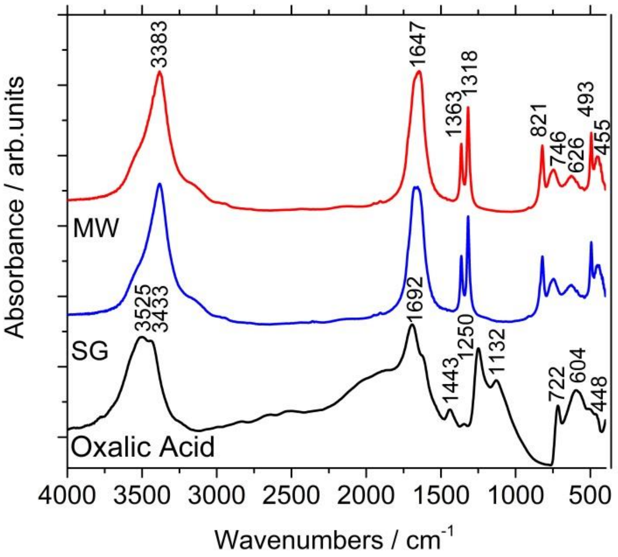

Figure S1: FTIR spectra of SG and MW samples after photocatalytic test compared to commercial oxalic acid.

Figure S2 shows the UV–vis spectra of pristine SG ZnO and MW ZnO samples. No visible light absorption (in line with literature data) or significant differences can be depicted for sol–gel ZnO and microwave ZnO. In addition, E_g values obtained by the Tauc method are larger (>3.1 eV) than those of manganese-doped samples. Having in view the photocatalytic application of the synthesized materials, namely the oxalic acid degradation under solar light irradiation, the manganese-modified samples seem to have a higher applicative potential than pristine ZnO samples.

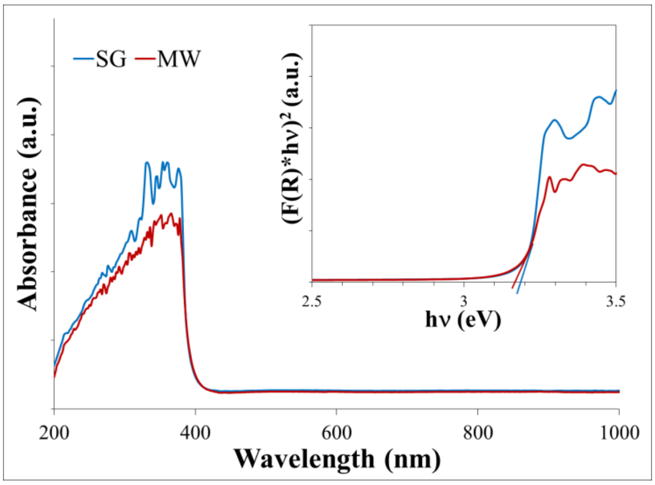

Figure S2: Comparative UV–vis spectra of SG ZnO and MW ZnO; the inset illustrates the Tauc representation for SG ZnO and MW ZnO samples.
